# Regulatory mechanisms for Snail protein stability: ubiquitin–proteasome system and chaperone-mediated autophagy

**DOI:** 10.1038/s12276-026-01667-6

**Published:** 2026-02-19

**Authors:** Minju Kim, Keun-Seok Hong, Taeyoung Kim, Ki-Jun Ryu, Jiyun Yoo

**Affiliations:** 1 Division of Applied Life Science (Brain Korea 21 Four), Research Institute of Life Sciences, Gyeongsang National University, Jinju, Republic of Korea; 2 Department of Biochemistry and Convergence Medical Science, Institute of Medical Science, College of Medicine, Gyeongsang National University, Jinju, Republic of Korea; 3 Division of Life Science, College of Natural Sciences, Gyeongsang National University, Jinju, Republic of Korea

**Keywords:** Metastasis, Post-translational modifications

## Abstract

Snail (*SNAI1*), a central transcription factor driving epithelial–mesenchymal transition (EMT), is pivotal in cancer metastasis and tissue remodeling. Owing to its labile nature, Snail activity is tightly controlled by post-translational modifications that dictate its stability. Here this review summarizes how the ubiquitin–proteasome system orchestrates Snail degradation through coordinated phosphorylation and ubiquitination, mediated by diverse E3 ligases and regulated by kinases, acetyltransferases and deubiquitinases. These mechanisms dynamically adjust Snail levels in both the cytoplasm and nucleus, thereby modulating EMT outcomes. In parallel, emerging studies reveal that chaperone-mediated autophagy (CMA) provides an additional layer of regulation. Through recognition of KFERQ-like motifs, CMA selectively directs cytoplasmic Snail to lysosomes for LAMP2A-dependent degradation, functioning as a quality control system. Impairment of CMA leads to nuclear accumulation of Snail, enhancing its EMT-inducing and prometastatic potential. Together, the ubiquitin–proteasome system and CMA represent complementary, context-dependent axes that maintain Snail homeostasis. Their disruption facilitates EMT activation and metastatic progression. By integrating recent findings, this review highlights the dual degradative control of Snail and its implications for cancer biology, providing a conceptual framework for therapeutic approaches aimed at restoring degradative balance and limiting metastasis.

## Dual control mechanisms of Snail in cancer progression

Cancer can spread in the body through a process called metastasis, which is often linked to a change in cell behavior known as epithelial–mesenchymal transition (EMT). A protein called Snail plays a key role in EMT by helping cancer cells become more mobile and invasive. Researchers are trying to understand how Snail is controlled because it could lead to new cancer treatments. Snail is usually broken down by the cell’s waste disposal system, the ubiquitin–proteasome system. This involves tagging Snail with a small protein called ubiquitin, which marks it for destruction. However, Snail can also be degraded through another process called chaperone-mediated autophagy, where it is transported to lysosomes for breakdown. This study found that when Snail avoids these degradation pathways, it accumulates and promotes cancer spread.

This summary was initially drafted using artificial intelligence, then revised and fact-checked by the author.

## Introduction

Metastasis is a key step in cancer progression and a major determinant of patient prognosis^1,2^. A critical early event in this process is epithelial–mesenchymal transition (EMT), during which epithelial cells acquire mesenchymal properties, including increased motility and invasiveness^3,4^. EMT allows tumor cells to escape from the primary lesion and initiate metastasis^5,6^. The transcription factor Snail plays a central role in this transition by repressing epithelial adhesion molecules and promoting mesenchymal gene expression^7,8^. Through these mechanisms, Snail facilitates EMT by promoting cell separation, migration and invasion. Snail overexpression is associated with increased tumor aggressiveness, a higher risk of metastasis and recurrence and poor prognosis in various carcinomas, including breast cancer^9,10^. Therefore, the tight regulation of Snail protein expression and stability is essential under normal physiological conditions^11,12^. Understanding the mechanisms underlying Snail degradation is therefore critical for developing therapeutic strategies aimed at inhibiting the EMT and controlling cancer metastasis^5,10,13,14^.

Snail is a highly labile transcription factor and cells exert tight control over EMT activation by rapidly degrading it^11^. The ubiquitin–proteasome system (UPS) is the best-characterized pathway for Snail degradation^13^. In this pathway, phosphorylation of specific residues by glycogen synthase kinase 3 beta (GSK-3β) enables Snail to be recognized by E3 ubiquitin ligases, such as beta-transducin repeat-containing protein (β-TrCP), leading to its ubiquitination and subsequent proteasomal degradation. Notably, Snail contains two major GSK-3β phosphorylation sites^12^. Phosphorylation at the first site unmasks a nuclear export signal, facilitating the translocation of Snail from the nucleus to the cytoplasm. Phosphorylation at the second site creates a destruction motif that enhances recognition by β-TrCP and promotes ubiquitination. Through this mechanism, cytoplasmic Snail is efficiently targeted for degradation by various E3 ligases, whereas nuclear-localized Snail is relatively protected from degradation and is thus more stable.

The subcellular localization of Snail is a critical determinant of its protein stability^13^. Kinases, such as GSK-3β, p21-activated kinase 1, large tumor suppressor kinase 2 (Lats2) and protein kinase D1 (PKD1), modulate Snail’s intracellular distribution, thereby influencing its degradation dynamics^9,13,15,16^. Spatial regulation has long been considered a central mechanism controlling Snail activity. However, accumulating evidence challenges this notion. Nuclear-resident E3 ubiquitin ligases, such as F-box and leucine-rich repeat protein 5 (FBXL5), peptidylprolyl isomerase-like 2 (PPIL2) and tripartite motif-containing 50 protein (TRIM50), mediate the ubiquitination of Snail within the nucleus, and nuclear proteasome complexes actively participate in its degradation^17–19^. These findings necessitate a revision of the previous model, in which nuclear localization is thought to confer protection from proteasomal turnover.

While Snail predominantly functions as a nuclear transcriptional repressor, it is also detected in the cytoplasm under various physiological and pathological conditions. The cytoplasmic pool of Snail may display differential stability depending on cellular context and the involvement of chaperones or signaling pathways. Thus, its turnover cannot be solely attributed to ubiquitination or proteasomal degradation. This implies that Snail degradation is modulated through multiple parallel mechanisms, possibly influenced by its subcellular localization. In this Review, we first summarize the general degradation mechanisms of Snail via the UPS and then discuss an emerging alternative route—chaperone-mediated autophagy (CMA)—as a context-dependent regulator of Snail stability.

## Ubiquitination and proteasomal degradation of snail protein

### Ubiquitination-dependent regulation of Snail protein stability

Snail is a transcription factor primarily functioning in the nucleus. Maintaining proper protein stability and nuclear localization is crucial for effectively performing its biological role. These processes can be regulated through various post-translational modifications (PTMs) of Snail. Although phosphorylation by kinases is a well-documented mechanism, ubiquitination by E3 ubiquitin ligases has also been widely reported.

Ubiquitination is a process in which a small 8.6-kDa protein, ubiquitin, is covalently attached to specific residues on a target protein. This process involves three key enzymes: the ubiquitin-activating enzyme (E1), ubiquitin-conjugating enzyme (E2) and ubiquitin ligase (E3)^20^. E1 activates ubiquitin by forming a high-energy thiol ester bond between the C-terminal Gly residue of ubiquitin and the Cys residue of E1 in an adenosine triphosphate-dependent manner. E2 then receives the activated ubiquitin from E1 through the formation of a thiol ester bond between its Cys and Gly residues. Although several E2 enzymes can directly transfer ubiquitin to substrate proteins, most transfer ubiquitin to the E3 ubiquitin ligase for the next process^21^. E3 ubiquitin ligases can be broadly classified into four families based on the structural domains that interact with the E2 enzymes: HECT, RING, U-box and RBR. Compared to E1 and E2 proteins, E3 ligases are more numerous and exhibit high specificity for recognizing substrate proteins^22^. Therefore, the function of selecting proteins to be ubiquitinated is performed by E3 proteins. These ligases facilitate the attachment of ubiquitin, which is transferred from E2 to lysine residues on substrate proteins or, in some cases, to serine, threonine or cysteine residues^21^.

Ubiquitination can be classified based on the link between ubiquitin and its substrate proteins. Mono-ubiquitination involves the attachment of a single ubiquitin molecule to one residue of a substrate protein. Multi-ubiquitination occurs when multiple ubiquitin molecules are linked to different residues on the substrate protein, rather than a single residue. Polyubiquitination refers to the process in which one ubiquitin molecule is conjugated to a substrate, additional ubiquitin is sequentially attached to residues within the first ubiquitin and the process is repeated to form a polyubiquitin chain^23–25^. Polyubiquitination can also be classified based on the specific residue to which ubiquitin is linked, either the N-terminal methionine (Met) or one of the seven lysine residues (K6, K11, K27, K29, K33, K48 or K63)^26^. This classification is important because the regulatory mechanisms of substrate proteins differ depending on how ubiquitin is linked, which, in turn, alters protein function^27^. For instance, the K48 linkage, the most commonly reported type, induces substrate degradation via the 26S proteasome^28^, whereas the K63 linkage is primarily involved in processes such as degradation via autophagy, cytokine signaling and DNA damage repair^29^. Although less frequently reported, other linkages have distinct roles: K6 linkage is associated with the DNA damage response^30^; K11 linkage regulates the cell cycle, membrane trafficking and innate immune response^31^; and K27 linkage is linked to protein secretion, DNA damage repair and the mitochondrial damage response^32–34^. Furthermore, K27, K29 and K33 linkages regulate the innate immune response by attaching to substrate proteins^35–37^. Finally, Met linkage inhibits interferon signaling while activating nuclear factor kappa-light-chain-enhancer of activated B cells (NF-κB) signaling^38^. Thus, ubiquitination serves as a precise regulatory mechanism that controls cellular functions based on how specific proteins are linked by various E3 ubiquitin ligases.

In addition, the intracellular expression and stability of Snail proteins are regulated by ubiquitination. In particular, there have been many reports on proteasomal degradation by K48-linked polyubiquitination, the first of which was an F-box protein called β-TrCP^39^. β-TrCP participates in the Skp1–cullin1–F-box protein (SCF) complex, which acts as an E3 ubiquitin ligase complex and acts as a substrate adapter for Snail protein^13^. Cullin1, which acts as a scaffold, can bind to RBX1 with a RING domain that interacts with E2 enzymes; the ubiquitin possessed by E2 enzymes is linked to Snail through this complex^13,40^. In summary, β-TrCP increases K48-linked polyubiquitination of Snail, inducing proteasomal degradation and thereby participating in the stability of Snail^13^. Similar to β-TrCP, several F-box proteins, such as FBXL14^41,42^, FBXO11^43^, FBXL5^17,44^, FBXO45^45^, FBXW7^46,47^, FBXO31^48^ and FBXO22^49^, which form the SCF complex and target Snail, regulate the stability by inducing K48-linked polyubiquitination in various cancers, including breast, gastric,and lung cancers. By contrast, SPRY domain-containing SOCS box protein 3 (SPSB3) is a SOCS box protein that binds to cullin5, which acts as a scaffold protein^50^. SPSB3 is a component of the E3 ligase complex that acts as a substrate adapter and targets Snail and induces K48-linked polyubiquitination in esophageal squamous cell carcinoma^51^. In addition, the E3 ubiquitin ligases tripartite motif-containing 21 (TRIM21)^52,53^, TRIM50^19^, and membrane-associated ring-ch-type finger 2 (MARCH2)^54^ have a RING domain, and PPIL2^18^ and carboxy-terminus of Hsc70-interacting protein (CHIP)^55^ have a U-box domain. Finally, an HECT domain E3 ubiquitin protein ligase 1 (HECTD1)^56^ has an HECT domain. All of these can bind to Snail and induce K48-linked polyubiquitination and proteasomal degradation, which are involved in the downregulation of Snail protein expression. They directly interact with E2 enzymes through the RING, U-box and HECT domains, simultaneously bind to Snail and perform ubiquitination as E3 ligases.

Snail is regulated by E3 ligases not only in the cytoplasm but also in the nucleus. It functions as a transcription factor that translocates from the cytoplasm to the nucleus via a nuclear localization signal^57,58^. Nuclear-translocated Snail can colocalize with E3 ubiquitin ligases, such as FBXL5^17^, FBXW7^46^ and PPIL2^18^ in the nucleus and induce K48-linked polyubiquitination. TRIM50^19^ binds to Snail in both the cytoplasm and nucleus and induces proteasomal degradation, thereby decreasing protein stability. As proteasomes found in eukaryotic cells exist in both the cytoplasm and nucleus, it is possible that Snail ubiquitinated by the above E3 ligases is degraded after translocation to the cytoplasm or in the nucleus^59^.

By contrast, E3 ubiquitin ligases increase the stability and expression of Snail. Pellino-1 is an EMT-inducing factor and its expression is increased in patients with lung cancer. Pellino-1 binds to Snail and induces K63-linked polyubiquitination, thereby inhibiting proteasomal degradation of Snail and increasing its stability^60^. In breast and ovarian cancers, an E3 ligase called mouse double minute 2 (MDM2) does not directly regulate Snail, but can induce EMT by increasing the mRNA expression of Snail by inducing the activation of upstream NF-κB/p65^61^ or Smad2/3^62^ transcription factors. Similarly, the E3 ubiquitin ligase MARCH7 is highly expressed in patients with endometrial cancer and is associated with poor prognosis because it increases the expression of Snail^63^. This suggests that E3 ubiquitin ligases are not only involved in the regulatory mechanisms that reduce Snail stability but also participate in mechanisms that increase Snail expression.

Other PTMs are also involved in the process by which E3 ligases act on Snail. In particular, there are many reports related to phosphorylation, which is the most common PTM in Snail. For example, the serine/threonine kinase GSK-3β interacts with various E3 ligases and determines the stability of Snail. First, in the case of β-TrCP, GSK-3β phosphorylates Snail in the nucleus, allowing its translocation into the cytoplasm. Subsequently, another motif in Snail must be phosphorylated in the cytoplasm for β-TrCP to properly recognize and bind to Snail^13^. Kinases reported to be upstream regulators of GSK-3β include casein kinase 1 (CK1) and dual-specificity tyrosine (Y)-phosphorylation regulated kinase 2 (DYRK2). CK1 phosphorylates Serine104/Serine107 in Snail^64^ and DYRK2 phosphorylates Serine104 in Snail^65^. These phosphorylated residues can be recognized and bound by GSK-3β, which can effectively induce phosphorylation of the Serine96/Serine100 motif of Snail and then, as described above, β-TrCP induces proteasomal degradation of Snail. In addition, FBXO31 in gastric cancer^48^, FBXO22 in breast cancer^49^ and SPSB3 in esophageal squamous cell carcinoma^51^ decrease the stability of Snail by depending on phosphorylation by GSK-3β.

There are also E3 ligases that are regulated by kinases through pathways independent of GSK-3β. In the case of FBXO11, PKD1^43^ and AMPK^66^ induce the phosphorylation of Serine11 in Snail. Thus, FBXO11 increases the recognition and binding of Snail and its ubiquitination. In the case of FBXL14, the interaction with Snail increases as LBK1 expression increases, inducing the proteasomal degradation of Snail and reducing the metastasis of pancreatic cancer cells^42^. In the case of atypical protein kinase C (aPKC), it can increase ubiquitination by β-TrCP in a GSK-3β-independent manner by phosphorylating Serine249 in Snail^67^.

Conversely, some kinases interact with E3 ligases to decrease Snail ubiquitination, thereby increasing its stability. In breast cancer, PTK6 expression is associated with a poor prognosis. PTK6 inhibition decreases Snail protein stability^68^, which ultimately confirms that PTK6 inhibition increases the association between Snail and the E3 ligase MARCH2, leading to its proteasomal degradation^54^. In the case of FBXL5, which induces Snail ubiquitination in the nucleus, phosphorylation of Snail threonine 203 by Lats2 in the nucleus inhibits its binding to FBXL5, thereby increasing Snail stability^17^. The tyrosine kinase PYK2 phosphorylates Snail’s tyrosine 163, thereby inhibiting its binding to CHIP and reducing ubiquitination^69^, and IKBKE phosphorylates Snail’s serine 165, thereby inhibiting its binding to β-TrCP^70^. In ovarian cancer, the phosphorylation of Serine107 of Snail by p38 prevents DYRK2 from binding to Snail and inducing phosphorylation. This blocks the subsequent action of GSK-3β, ultimately inhibiting proteasomal degradation by β-TrCP^71^. Furthermore, in pancreatic cancer, ERK3 inhibits Snail protein degradation solely by binding to FBXO14, without phosphorylating Snail^72^.

There have been reports on its association with other PTM-related enzymes, including acetyltransferases. A protein called BRD4 increases the progression and metastasis of cancer cells in gastric cancer. This is because when an acetyl transferase called CREB-binding protein causes acetylation of K146 and K187 of Snail, BRD4 binds to it, preventing the binding of β-TrCP and FBXL14, which had previously targeted and degraded Snail, thereby increasing the stability of Snail^73^. This suggests that E3 ubiquitin ligases do not work alone in regulating Snail but also interact with multiple PTM-related enzymes.

**Fig. 1:**
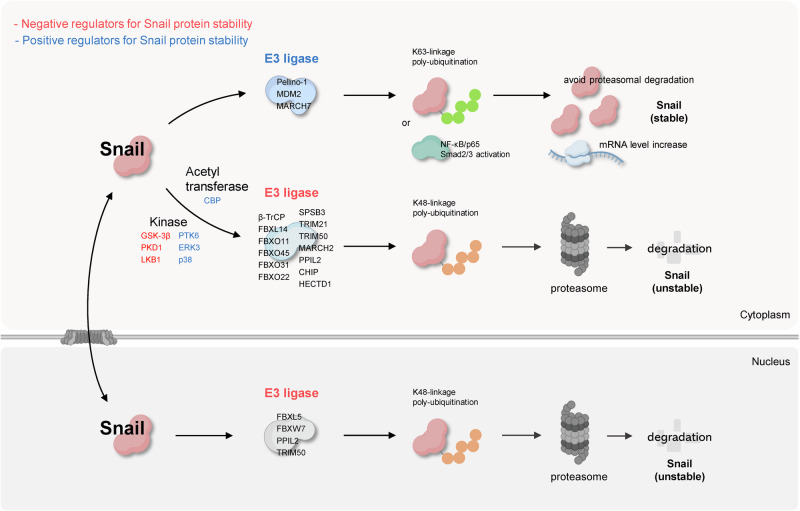
Ubiquitination-dependent regulation of Snail protein stability. Snail is a highly labile protein that undergoes rapid degradation via the proteasome. Multiple E3 ubiquitin ligases promote K48-linked polyubiquitination of Snail, targeting it for cytoplasmic and nuclear proteasomal degradation. By contrast, certain E3 ligases inhibit proteasomal turnover and enhance Snail stability. Additional PTMs, including phosphorylation and acetylation, modulate the activity of E3 ligases and thereby fine-tune Snail protein stability.

Ubiquitination by E3 ubiquitin ligase acts as a major mechanism that determines the induction of proteasomal degradation of Snail and is greatly involved in the intracellular stability of Snail. Various E3 ligases contribute to the progression and metastasis of tumors by regulating Snail depending on tissue specificity or intracellular signaling. The function of E3 ligases that induce K48-linkage polyubiquitination is to reduce the cytoplasmic and nuclear stability of Snail, thereby inhibiting the progression and metastasis of cancer. Conversely, E3 ligases that induce K63-linkage polyubiquitination have been confirmed to induce the malignancy of cancer by increasing the stability of Snail. In addition, it has been reported that the ubiquitination process of Snail closely interacts with other PTMs by kinase or acetyl transferase. The complexity and specificity of these regulatory mechanisms are essential for understanding the protein stability network centered around Snail, and strategies to inhibit or activate specific E3 ligases or related enzymes based on this may be promising approaches for the development of future EMT inhibition and anticancer therapeutics (Fig. 1).

Given that Snail protein stability is governed by multiple PTMs, it is also important to consider the upstream oncogenic signaling pathways that regulate these PTM-dependent processes. Several cancer-associated cues directly influence the phosphorylation and ubiquitination axis of Snail. For example, Wnt/β-catenin and PI3K–AKT signaling inhibit GSK-3β activity, thereby blocking phosphorylation and β-TrCP-mediated ubiquitination, ultimately enhancing Snail stability^13^. Growth factor signaling, including EGFR activation, stimulates p38 MAPK, which phosphorylates Snail at Ser107 to block DYRK2-mediated priming phosphorylation and subsequently suppresses GSK-3β–β-TrCP-dependent ubiquitination, thereby enhancing Snail stability^71^. By contrast, p53 acts as a suppressive cue by promoting the activity of ubiquitin ligases such as MDM2 and FBXO11 or by maintaining GSK-3β activity, collectively sensitizing Snail to proteasomal turnover^74^. Additional oncogenic pathways—including TGFβ–TAK1–MAPK signaling, NF-κB-mediated inflammatory signaling, Hippo pathway output and various stress-responsive signaling cascades—have also been shown to directly regulate the PTM machinery governing Snail stability by modulating its associated kinases, E3 ubiquitin ligases and deubiquitinases (DUBs)^3,15,43,66,67,69,75^. Collectively, these upstream signals illustrate how Snail functions as an integrative hub that translates diverse oncogenic inputs into PTM-dependent stabilization, providing essential context for understanding the PTM-based regulatory mechanisms of Snail.

### Deubiquitination of Snail protein

Deubiquitination is an enzymatic process in which the ubiquitin moieties attached to proteins are removed, thereby preventing their degradation or modulating their functional activity^76^. This process is mediated by DUBs, which cleave polyubiquitin chains from substrate proteins, suppress proteasomal degradation and enhance cell stability^76,77^. Typically, K48-linked polyubiquitination acts as a signal for proteasomal degradation; when removed by DUBs, the substrate protein is stabilized^75^. Beyond this classical mechanism, DUBs also influence substrates via crossregulation with other PTMs, act on K63-linked polyubiquitin chains or perform nonenzymatic functions (for example, scaffolding through complex formation) independent of catalytic activity^78,79^. Therefore, it is essential to characterize precisely how DUBs modulate substrate stability.

More than 100 deubiquitinating enzymes (DUBs) have been identified in humans and are broadly classified into five major families based on their structural similarity and catalytic domains: (1) ubiquitin-specific proteases (USPs), (2) ubiquitin C-terminal hydrolases, (3) ovarian tumor proteases, (4) Machado–Josephin domain-containing proteases and (5) JAB1/MPN/Mov34 metalloenzymes (JAMM/MPN^+^)^26,80^. USPs constitute the largest DUB family in humans, comprising >50 members. DUBs recognize their substrates via several distinct mechanisms^81^. First, they often act specifically by recognizing the type of ubiquitin chain linkage, such as K48-linked chains, which typically signal proteasomal degradation^82^. The removal of these chains by DUBs leads to substrate stabilization. Second, DUBs selectively interact with substrates by directly recognizing specific domains or structural motifs within substrate proteins that serve as binding interfaces^24^. Third, some DUBs utilize ubiquitin-binding domains or interact through adapter proteins or multiprotein complexes to recognize their substrates more efficiently^81^. This implies that DUB–substrate interactions may not occur in vitro, but may be context dependent and observable only under specific cellular or in vivo conditions.

Various PTM-related proteins have been identified in studies aimed at identifying novel regulators of Snail protein stability. Among these, deubiquitinating enzymes (DUBs) have emerged as potential therapeutic targets^83^. Multiple human DUBs increase Snail protein levels, indicating their potential as novel modulators of Snail stability^84^. However, importantly, some DUBs may regulate Snail stability through mechanisms that are independent of direct substrate recognition, which warrant further validation^24^.

If Snail protein stability is increased by a DUB, it is necessary to verify whether Snail acts as a direct substrate for the DUB. Using BioGRID 4.4 to explore the interactions between Snail and DUBs, it was observed that many DUBs potentially bind directly to Snail. In one study, 23 DUBs were found to interact with Snail via coimmunoprecipitation and were confirmed to affect Snail stability through western blotting analysis^85^. However, although direct binding is observed, not all DUBs regulate Snail stability. This is because, in addition to the canonical mechanism of removing K48-linked polyubiquitin chains to prevent proteasomal degradation, DUBs can also regulate substrate protein functions or subcellular localization via other PTMs or act as adapter proteins in multiprotein complexes^24,83^. Thus, direct binding may not lead to changes in Snail stability^85^. To determine whether DUBs that bind to Snail functionally regulate its stability as a substrate, ubiquitination assays should be conducted. In addition, Snail polyubiquitination levels should be evaluated by inhibiting DUB catalytic activity or altering DUB protein expression.

Furthermore, there are cases in which a DUB that does not regulate Snail stability under normal conditions may become functional in specific cellular contexts^86,87^. Cellular conditions present within certain cancer types or in highly metastatic states may involve numerous signaling pathways and PTMs that can influence the interaction between DUBs and Snail. For example, one study demonstrated that phosphorylation of DUB3 by CDK4/6 enhanced the interaction between DUB3 and Snail, leading to increased Snail stability^88^. Another study showed that the phosphorylation of Snail by MSK1 promoted its binding to USP5, thereby stabilizing the Snail protein^89^. These findings suggest that DUB–substrate interactions may occur through various mechanisms, including PTM-dependent recognition and changes in the catalytic activity or substrate affinity of DUBs^24^. Therefore, importantly, DUB–Snail interactions may not always occur under basal conditions but may be induced under specific cellular or pathological states^83^.

In summary, it is necessary to determine whether DUB proteins regulate the stability of Snail not only through deubiquitination but also via other specific mechanisms they may exert within the cell. In cases where direct binding contributes to Snail stabilization, it should be clarified whether this occurs through the catalytic deubiquitination activity of DUB. In addition, whether such direct interactions are condition dependent and triggered by specific environmental factors must be considered.

## CMA-dependent regulation of snail protein

In addition to proteasomes, cells utilize autophagy as an alternative route for protein degradation. CMA is a pathway that selectively transports specific proteins to lysosomes for degradation; its mechanism is distinct from that of macroautophagy and microautophagy^90^. CMA confers selectivity using the KFERQ motif or a similar sequence present in target proteins as a recognition signal^91^. Proteins with the corresponding sequence bind to HSC70 and are delivered to the lysosomal membrane receptor lysosome-associated membrane protein 2A (LAMP2A) in cooperation with cochaperone proteins^92^. Subsequently, the partially unfolded substrate is translocated to the lysosomal lumen and degraded by various hydrolases^93^. Through this process, CMA contributes to cellular homeostasis and metabolic balance by selectively removing cytoplasmic proteins^94^.

Snail functions as a substrate for CMA^95^. Cytoplasmic Snail undergoes CMA-dependent degradation in breast cancer cells. In luminal-type breast cancer cells, which represent less aggressive subtypes, Snail is mostly localized in the cytoplasm and is degraded via CMA. By contrast, in aggressive triple-negative breast cancer cells, Snail accumulates in the nucleus and evades CMA-mediated degradation. These findings suggest that the intracellular localization of Snail determines its degradation by CMA, resulting in differences in Snail stability and EMT-inducing ability.

Snail degradation via CMA depends on its transport and translocation into lysosomes, which require the LAMP2A receptor. Furthermore, the presence of a KFERQ motif or similar sequences in the protein allows it to be recognized by HSC70 and enter the CMA pathway^91,95^. Certainly, treatment of cells with lysosomal inhibitors (for example, NH₄Cl, leupeptin) significantly increases Snail protein levels, and immunofluorescence analysis confirmed colocalization of Snail with lysosomal markers (LAMP1). Furthermore, knockdown of LAMP2A disrupts the lysosomal trafficking of Snail, impairing its CMA-mediated degradation and resulting in increased protein stability. Overall, these findings provide strong evidence that Snail undergoes LAMP2A-dependent lysosomal degradation and acts as a bona fide substrate for CMA^95^.

Sequence analysis and predictions using the KFERQ finder program revealed that several KFERQ-like motifs exist in the Snail protein, and functional mutational analysis confirmed that the sequence located at amino acids 58–62 was essential for CMA recognition^91,95,96^. The 58AAAA-Snail mutant, in which the residue in this region was substituted with alanine, lost its binding to HSC70 and did not interact with LAMP2A, thereby blocking transport to lysosomes. Consequently, this mutant form of Snail evaded lysosomal degradation and accumulated in cells, resulting in significantly elevated protein levels to with the wild type. These results demonstrate that Snail functions as a typical CMA substrate and that the KFERQ-like motif in amino acids 58–62 is essential for CMA-dependent degradation. In summary, cytoplasmic Snail is recognized by HSC70 cells through a specific KFERQ-like motif and transported to the lysosome via LAMP2A for degradation. This CMA-dependent degradation pathway functions independently of the UPS and represents a critical mechanism for regulating Snail protein stability and activity^95^.

Functional evidence indicates that the accumulation of Snail, which escapes CMA-mediated degradation, enhances EMT and metastatic potential^10,95^. Breast cancer cells (MCF-7) overexpressing 58AAAA-Snail exhibited notable changes in their mesenchymal morphology compared to wild-type or control cells. Regarding EMT marker expression, wild-type Snail-expressing cells showed no substantial differences between epithelial and mesenchymal markers, whereas cells expressing a CMA-resistant Snail mutant showed a notable increase in mesenchymal markers and a decrease in epithelial markers. Certainly, the cells exhibited significantly enhanced migration and invasive capabilities, and in a mouse lung metastasis model, they formed significantly more metastatic nodules than wild-type controls^95^. These results suggest that the CMA pathway acts as a physiological regulatory mechanism that contributes to Snail-mediated EMT and suppression of metastasis.

Collectively, these findings support the hypothesis that CMA functions as a physiological brake in Snail-driven EMT and metastasis. When Snail evades this pathway, it accumulates and promotes malignant progression. Therefore, CMA may represent a critical regulatory axis for Snail stability and may serve as a promising therapeutic target for limiting EMT and metastatic dissemination.

## Conclusions

**Fig. 2:**
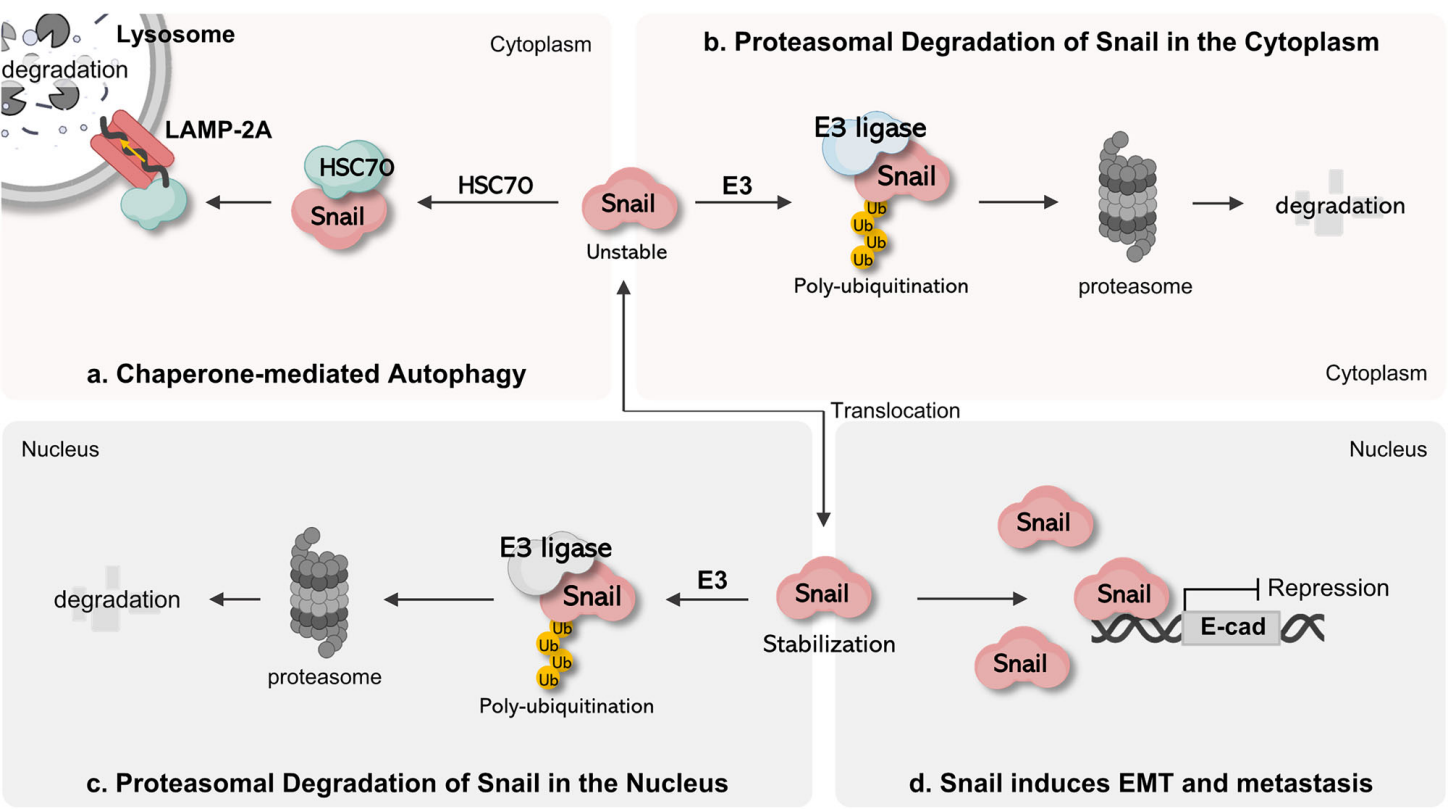
Degradation pathways of Snail and its role in EMT induction. **a** CMA: cytoplasmic Snail protein is recognized by HSC70 and selectively transported into lysosomes via LAMP2A for degradation. **b** Cytoplasmic ubiquitin–proteasome pathway: Snail undergoes polyubiquitination by E3 ligases and is subsequently degraded by cytoplasmic proteasomes. **c** Nuclear ubiquitin–proteasome pathway: nuclear-localized Snail, although functionally stabilized, can be ubiquitinated by nuclear E3 ligases and degraded within the nuclear proteasome system. **d** EMT and metastasis induction: nuclear Snail represses E-cadherin (E-cad) transcription, thereby driving EMT, enhancing tumor cell invasiveness and promoting metastatic progression.

The discovery that Snail is selectively degraded in lysosomes via CMA provides new insights into the mechanisms underlying EMT regulation^95^. Although Snail degradation has primarily been studied through the UPS, recent studies have demonstrated that CMA plays an independent and essential role in the regulation of Snail protein stability and function^13,95^. Specifically, CMA selectively removes cytoplasmic Snail, thereby suppressing its nuclear accumulation and blocking EMT induction, thus acting as a ‘biological safety device’ (Fig. 2).

Conversely, when Snail accumulates in the nucleus or when CMA activity is suppressed, EMT is induced and tumor cell invasiveness and metastatic potential are markedly increased. This suggests that the spectrum of substrate proteins selected based on the physiological state of the cell, rather than the level of CMA activity itself, may have a critical impact on cancer progression, particularly at the metastatic stage^94,97^. In other words, the targets recognized and degraded by CMA may vary depending on intracellular signaling pathways, acquired protein modifications (PTMs) and molecular stress states, and such context-dependent changes in substrate selectivity may act as key regulatory factors in the induction of EMT and acquisition of malignant phenotypes in tumor cells.

The complementary and conditional interplay between CMA and UPS in regulating Snail degradation constitutes a dual degradation axis, which may serve as a foundation for the development of novel therapeutic strategies to control metastasis and malignancy in cancer. In particular, the Snail–CMA axis has the potential to be a promising molecular mechanism for the development of EMT inhibitory therapies. In the future, strategies that indirectly increase CMA sensitivity by modulating CMA activity or controlling Snail subcellular localization may offer novel avenues for antimetastatic therapies.
